# Testicular Signet-Ring Cell Metastasis from a Carcinoma of Unknown Primary Site: A Case Report and Literature Review

**DOI:** 10.1155/2016/7010173

**Published:** 2016-07-18

**Authors:** Aristomenes Kollas, George Zarkavelis, Anna Goussia, Aikaterini Kafantari, Anna Batistatou, Zoi Evangelou, Eva Sintou, Nicholas Pavlidis

**Affiliations:** ^1^Department of Medical Oncology, University Hospital of Ioannina, 45500 Ioannina, Greece; ^2^Department of Pathology, University Hospital of Ioannina, 45500 Ioannina, Greece; ^3^Department of Cytology, University Hospital of Ioannina, 45500 Ioannina, Greece

## Abstract

Signet-ring cell carcinoma is a highly malignant adenocarcinoma consisting of cells characterized as cytoplasmic vacuoles filled with mucin. The most common primary location of this type of cancer is the stomach, but it may also be found in other organs such as prostate, testis, bladder, ovaries, or colon. To date, metastatic signet-ring cell carcinoma of unknown primary (CUP) site to the testis is an extremely rare entity in daily practice. Reviewing the literature, we have been able to detect only three cases of testicular metastases from CUP, two with histological diagnosis of a signet-ring cell carcinoma and one with an adenocarcinoma. In this short paper, we report a case of a 56-year-old man who presented to our Department with testicular mass and ascites. Following a standard diagnostic approach no primary tumor could be identified. CUP was the final clinical diagnosis, histologically characterized as poorly differentiated adenocarcinoma with signet-ring cells involving the peritoneum and the testicular structures.

## 1. Introduction

CUP is a clinical syndrome, which is defined by the presence of metastatic disease without establishment of the primary site. Throughout the literature, the term occult is also used when referring to a type of malignancy with uncertain site of uncertain origin outcome, without definitive IHC findings and clinical manifestations. Its frequency is estimated about 3% to 5% of all malignancies and it is represented with various clinical and histologic characteristics. The natural history of the disease is characterized by a short time of symptoms and rapid dissemination of the disease. The diagnostic algorithm is based on patient's symptoms, clinical examination, laboratory findings, and imaging studies. A more favorable prognosis has been associated with lymph nodal disease, female sex, good performance status, normal LDH levels, and small number of metastatic sites [1, 2].

In order to identify the primary site, a thorough physical examination, a complete medical history, and basic laboratory tests such as complete blood count, serum biochemistry, chest X-ray, CT scans, mammography, and tumor markers should be performed [2, 3]. Accumulating data emphasize the limited role of PET/CT in diagnosing a probable primary site, mainly if head and neck cancer is suspected [4–6]. Basic IHC stains are used to increase the ability to identify the primary organ sites, such as CK7, CK20, chromogranin, synaptophysin, NSE, TTF-1, thyroglobulin, CDX-2, PSA, AFP, b-hCG, vimentin, S100, HMB 45, ER, or PR [7]. At the same time, more accurate methods such as Molecular Tumor Profiling technics (MTP) are available to help oncologists define the primary site [8]. The primary goal of medical oncologists is to rule out the presence of a potentially treatable or curable malignancy (i.e., germ-cell tumors, lymphomas, and breast cancer) [2].

Association of CUP with signet-ring histology is very rare, especially with the presence of testicular metastasis. We, therefore, introduce a case of a 56-year-old man, who presented to our Department with a testicular mass and ascites, without the presence of a primary site following extensive diagnostic work-up. Our final diagnosis was cancer of unknown primary.

## 2. Case Presentation

A 56-year-old male Caucasian, 60-pack-year smoker with a past medical history of sleep apnea presented as an outpatient with gradual abdominal distention. During the last 2 months he reported painless swelling of the right testis. Physical examination revealed ascites and right scrotal hard mass with enlarged testis. Complete blood count and biochemistry were normal, while serum CA 125 was increased (319 *μ*/mL). In November of 2015 he was admitted to the Oncology Department for further investigation.

**Figure 1:**
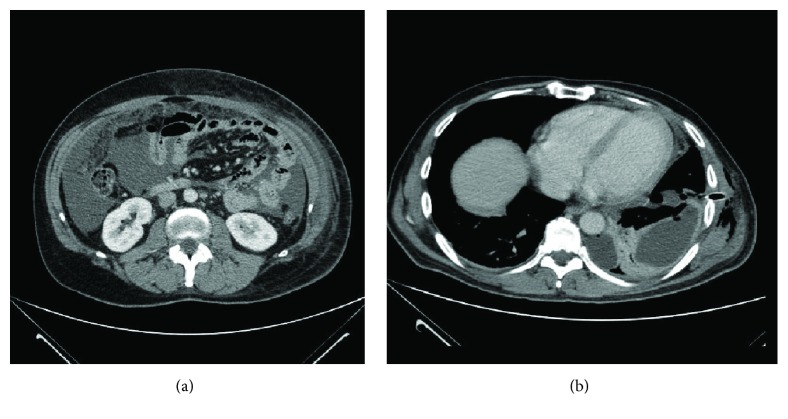
(a) At the time of diagnosis diffuse peritoneal fluid in the abdomen and peritoneal implants are presented through the CT scan. (b) Pleural effusion on the left (progressive disease) presented after the third cycle of Capecitabine/Oxaliplatin chemotherapy.

**Figure 2:**
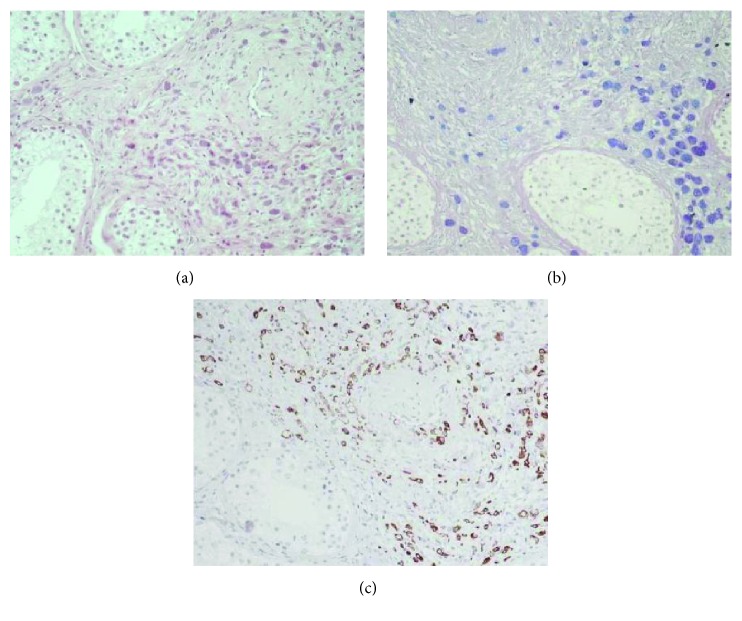
(a) The testicular parenchyma is infiltrated by neoplastic signet-ring cells (hematoxylin-eosin ×200). (b) Tumor cells exhibit positivity for mucin stains (arrows). (c) Immunohistochemically the tumor cells were positive for cytokeratin 20 (arrows).

Computed tomography of the thorax and abdomen revealed a minimal pleural effusion of the left hemithorax, diffuse peritoneal fluid in the abdomen, and peritoneal implants (Figure 1(a)). Since no solid literature data exist (apart from the sensitivity of PET/CT scan in hidden primaries mainly of head neck) no PET/CT scan was requested in our case. Upper and lower GI endoscopy revealed no abnormalities. Patient had a scrotal ultrasound imaging that revealed an enlarged right epididymis with small amount of fluid in the right side of the scrotum. Abdominal paracentesis revealed exudative fluid with neoplastic signet-ring cells indicative of metastatic adenocarcinoma. Gross evaluation of the tissue specimen revealed several poorly defined, whitish, and hard in consistency foci throughout the testicular parenchyma, the epididymis, and the spermatic cord. The tunicae surrounding the testis were thickened. Microscopical examination of multiple tissue sections taken from the grossly described foci showed the presence of a poorly differentiated carcinoma composed of signet-ring cells (Figure 2(a)). Perineural and neural invasion as well as vascular invasion were observed.

By histochemical stains (PAS, Alcian Blue) a large amount of mucin was demonstrated in the cytoplasm of tumor cells (Figure 2(b)). Immunohistochemically, the neoplastic cells were diffusely positive for cytokeratin 20 and EMA, focally positive for cytokeratin 7, CEA, and c-kit (CD117), and negative for PLAP, a-fetoprotein, CD30, inhibin, calretinin, PSA, p504S (AMACR), TTF-1, and Melan A (Figure 2(c)). The pathological diagnosis was in favor of a metastatic adenocarcinoma, probably of gastrointestinal origin. Tissue HER2 was negative.

Taking into consideration the aforementioned findings, the primary site could not be established and the case was classified as CUP. In November 2015 he started on systemic therapy consisting of Capecitabine and Oxaliplatin. Up till January 2015, he has received three cycles of the above regimen with good partial remission of his ascites and excellent drug toleration. However, just before the fourth cycle he developed right pleural effusion with accompanying moderate dyspnea (Figure 1(b)). Pleural fluid cytology was positive for metastatic adenocarcinoma with signet-ring cells.

A second-line regimen consisting of Doxorubicin, Cyclophosphamide, and Fluorouracil (DCF) was administered. Up till now, the patient has received six cycles of DCF with complete response of the disease on abdominal and thoracic CT scans as well as normalisation of serum CA 125 (6 *μ*/mL).

## 3. Discussion

Cancer of unknown primary remains a neoplastic entity usually with an aggressive natural history and poor outcome. At the time of patient presentation an extensive investigation is needed in order to identify the primary site. Failure to identify the primary site leads to the establishment of CUP diagnosis. Cisplatin based chemotherapy in combination with a taxane is the main recommended empirical regimen.

Histologically, CUP includes well and moderately differentiated adenocarcinomas, squamous cell carcinomas, neuroendocrine carcinomas, poorly differentiated carcinomas, and undifferentiated carcinomas. CUP is distinguished between favourable (nodal disease and neuroendocrine tumors) and unfavourable (splanchnic metastases) subsets [2]. CUP can also be presented as isolated effusion or peritoneal carcinomatosis [12, 13]. To date, few cases of signet-ring carcinomas of occult primary site with metastases in the testicles have been reported. In particular, there are two similar cases through the literature with poor prognosis despite the therapeutic efforts that have been made.

Although the testicles are considered to be an inhospitable environment for cancer cells due to the low temperature, rarely neoplastic cells are able to invade them through the systematic venous, lymphatic circulation, or direct tumor invasion [14]. Although testicular metastases from other solid tumors have been rarely described, it is known that prostate cancer is the commonest primary tumor with such a predilection [15, 16]. In this paper, we presented the fourth case of a CUP patient diagnosed with a metastatic scrotal lesion (see Table 1).

In 2004 Salesi et al. reported a case of a 62-year-old man who presented with dyspnea, while a testicular mass and lung metastases with pleural effusion were noted. The final diagnosis according to the IHC studies was occult gastrointestinal adenocarcinoma [16].

In 2008 Chimakurthi and Lalit reported a case of a 37-year-old man with a history of alcoholism and alcoholic liver disease, who presented with ascites and right scrotal swelling. Testicular biopsy revealed metastatic adenocarcinoma with signet-ring cell features of an unknown primary site [10]. It was in 2011 when Saredi et al. reported a case of a 77-year-old man complaining of right testicular swelling. Orchiectomy revealed metastasis from poorly differentiated neoplasm with signet-ring cells, while prostatic biopsy revealed a unilateral acinar prostatic adenocarcinoma. Despite detailed diagnostic investigations, no primary site was detected and the final diagnosis was CUP with testicular signet-ring metastasis [11].

As a take home message, it should always be kept in mind that the clinical differential diagnosis of testicular mass—apart from primary cancers such as germ-cell tumors or non-Hodgkin's lymphomas—metastatic lesions from various solid tumors must be ruled out.

## 4. Conclusion

To date, several cases of metastatic adenocarcinomas to the testicles with primary tumors in prostate, lung, stomach, colon, or kidney have been reported. However, the diagnosis of signet-ring cell metastases to the testis from an unknown primary carcinoma is very uncommon. Conclusively, oncologists have to take into account the case of occult primary testicular metastasis with signet-ring cells as an extremely rare but existing possibility.

## Figures and Tables

**Table 1:** Reported cases of CUP with testicular metastases.

Author	Patient	Presenting symptom	Disease extent	Histological findings
Salesi et al. 2004 [9]	62-year-old male	Dyspnea and left testicular mass	Multiple lung metastases, pleural effusion, mediastinal node involvement, brain metastasis, and testicular metastasis	Metastatic adenocarcinoma (testicular biopsy and VATS)

Chimakurthi and Lalit 2008 [10]	37-year-old male	Ascites and right scrotal swelling	Diffuse carcinomatosis involving most of the abdominal organs, bowel obstruction, and frozen retroperitoneum	Metastatic adenocarcinoma with signet ring cells (testicular biopsy and omental biopsy)

Saredi et al. 2011 [11]	77-year-old male	Right testicular swelling	Pulmonary metastases and peritoneal carcinomatosis	Signet ring adenocarcinoma (testicular biopsy) Acinar prostatic adenocarcinoma (prostatic biopsy)

Kollas et al. 2006 (present case)	56-year-old male	Ascites and right testicular swelling	Peritoneal, testicular, and pleural metastases	Poorly differentiated carcinoma composed of signet ring cells
